# Deep Learning-Based CT Segmentation of a Non-radiopaque Hydrogel Rectal Spacer in Prostate Radiotherapy

**DOI:** 10.7759/cureus.111686

**Published:** 2026-06-28

**Authors:** Hideharu Miura, Shuichi Ozawa, Masahiro Kenjo, Yuji Murakami

**Affiliations:** 1 Radiation Oncology, Hiroshima High-Precision Radiotherapy Cancer Center, Hiroshima, JPN; 2 Radiation Oncology, Hiroshima University, Hiroshima, JPN

**Keywords:** automatic segmentation, deep learning, organs at risk, prostate cancer, rectal spacer

## Abstract

Introduction: The study evaluated the feasibility and geometric accuracy of CT-based automatic segmentation of a non-radiopaque hydrogel rectal spacer using a commercial deep learning platform (OncoStudio) and determined whether accurate spacer delineation can be achieved without MRI while preserving the contouring quality of adjacent organs.

Methods: This retrospective study included 21 patients with localized prostate cancer who underwent external beam radiotherapy with a non-radiopaque hydrogel rectal spacer (SpaceOAR; Boston Scientific). CT-based automatic segmentation of the rectal spacer, prostate, rectum, and bladder was performed on the planning CT using OncoStudio (version 2.0; Oncosoft Inc.). Reference contours were manually delineated on the planning CT with reference to co-registered T2-weighted MRI. Geometric accuracy was assessed using the Dice similarity coefficient (DSC), 95th-percentile Hausdorff distance (HD₉₅), and mean surface distance (MSD).

Results: The mean DSC for the rectal spacer was 0.787 ± 0.073, with a mean HD₉₅ of 4.03 ± 1.83 mm and a mean MSD of 1.18 ± 0.63 mm. The adjacent organs showed high geometric agreement, with mean DSC values of 0.876, 0.890, and 0.963 for the prostate, rectum, and bladder, respectively. No systematic over- or under-segmentation was observed, and the MSD for all structures was below 2.5 mm.

Conclusions: CT-based automatic segmentation using OncoStudio provided clinically acceptable boundary delineation of a non-radiopaque hydrogel rectal spacer without requiring MRI, while maintaining the contouring quality of the surrounding organ. These findings suggest that CT-only automatic contouring can reduce spacer-specific MRI use and associated registration uncertainty in prostate radiotherapy.

## Introduction

External beam radiotherapy is the standard curative treatment for localized prostate cancer, but rectal toxicity often becomes the dose-limiting factor because of the close proximity of the anterior rectal wall. Absorbable hydrogel rectal spacers can create a temporary separation between the prostate and rectum, enabling safer dose escalation and reducing gastrointestinal toxicity. Randomized and prospective studies have demonstrated that these spacers improve rectal dosimetry and reduce grade ≥2 gastrointestinal toxicity [1,2]. These clinical advantages, however, depend on accurate and reproducible delineation of the spacer and surrounding organs at risk (OAR). In routine practice, manual contouring is labor-intensive and prone to interobserver variability, which may affect treatment planning and dosimetry [3]. Deep learning-based automatic segmentation has therefore been implemented to reduce contouring workload and improve consistency across observers and institutions [4].

Two hydrogel spacer formulations are in clinical use: a radiopaque spacer that is clearly visible on computed tomography (CT) and a non-radiopaque spacer that appears nearly isodense to the surrounding soft tissue, making it difficult to distinguish. Correspondingly, the radiopaque formulation shows higher Hounsfield units on CT, whereas the non-radiopaque formulation exhibits attenuation values similar to soft tissue [5,6]. T2-weighted magnetic resonance imaging (MRI) is the reference standard for delineating the non-radiopaque spacer. Previously reported CT-based deep learning segmentation of the non-radiopaque spacer has demonstrated limited clinical utility because of low geometric agreement and degraded contours of the adjacent prostate and rectum [7]. Consequently, transferring the spacer contour from MRI to the planning CT through image registration introduces spatial uncertainty into the planning geometry [8]. Direct delineation of the spacer on the planning CT would eliminate this registration-related error and extend applicability to patients who cannot undergo MRI. OncoStudio (Oncosoft Inc., Seoul, South Korea) is a commercial deep learning-based segmentation platform that includes rectal spacers as a CT-based segmentation target. Prior studies have demonstrated high geometric accuracy for the prostate, rectum, and bladder in patients without a spacer [9]. However, CT-based segmentation of a non-radiopaque hydrogel spacer using this platform has not been evaluated.

In this study, we evaluated the feasibility and geometric accuracy of CT-based automatic segmentation of a non-radiopaque hydrogel rectal spacer using OncoStudio and assessed whether accurate spacer delineation can be achieved without MRI while preserving the contouring quality of the prostate and adjacent OAR, including the rectum and bladder.

## Materials and methods

Patient characteristics

This retrospective study included 21 patients with localized prostate cancer who underwent external beam radiotherapy with a non-radiopaque absorbable hydrogel rectal spacer (SpaceOAR; Boston Scientific, Marlborough, MA) between June 2021 and March 2026 at the Hiroshima High-Precision Radiotherapy Cancer Center in Hiroshima, Japan. All 21 cases represented consecutive patients treated with the non-radiopaque spacer during this period at our institution. Inclusion criteria were localized prostate cancer treated with definitive external beam radiotherapy, successful implantation of a non-radiopaque hydrogel spacer, and availability of both planning CT and T2-weighted pelvic MRI suitable for treatment planning. Patients were excluded if imaging data were incomplete or if metallic implants in the femoral heads or pelvis caused substantial distortion of the CT or MRI images. This study was approved by the Institutional Review Board of Hiroshima University (approval number: E2017-947-02).

CT simulation and MRI acquisition

All patients underwent planning CT and pelvic MRI as part of the standard imaging protocol for treatment planning. Patients were instructed to maintain a full bladder and an empty rectum at the time of simulation. The planning CT scan was performed using an Optima CT580W (GE Healthcare, Milwaukee, WI, USA) at 120 kVp with a slice thickness of 2.5 mm while the patients were in the supine position. The pelvic MRI was performed on a 3.0-T scanner (Discovery MR750w; GE Healthcare, Milwaukee, WI, USA) using a T2-weighted fast spin-echo sequence (TR 4500 ms, TE 100 ms, slice thickness 2.5 mm, in-plane resolution 0.9 × 0.9 mm). In this sequence, the non-radiopaque hydrogel spacer appears as a hyperintense structure in the retroprostatic space and is clearly distinguishable from the surrounding soft tissue.

Reference contours

For each patient, the rectal spacer, prostate, rectum, and bladder were manually delineated on the planning CT scan using RayStation (RaySearch Laboratories, Stockholm, Sweden). MRI-CT image registration was performed using a rigid registration algorithm within RayStation, and all contours were delineated on the planning CT with reference to the co-registered T2-weighted MRI. Because the non-radiopaque hydrogel spacer is nearly isodense with surrounding soft tissue on CT, MRI served as the primary reference for spacer delineation. The rigid registration was performed by aligning the prostate and surrounding pelvic anatomy between CT and MRI, and registration quality was visually verified in all three orthogonal planes by the treating radiation oncologist for each case. Based on routine clinical practice, the residual misalignment of the prostate and spacer region was estimated to be within a few millimeters, and we acknowledge that this registration uncertainty is inherently incorporated into the reference contours used for evaluation.

Initial contouring of the prostate, rectum, bladder, and spacer was performed by three board-certified radiation oncologists with 5-15 years of experience in genitourinary radiotherapy, following our institutional contouring protocol. All reference contours were subsequently reviewed and, if necessary, adjusted and approved by a senior radiation oncologist specialized in prostate radiotherapy. Contours followed our institutional prostate radiotherapy protocol: the prostate was delineated as the clinical target volume (CTV) including the entire gland, the rectum was contoured as the entire rectal lumen and anal canal within approximately 2 cm cranio-caudally above and below the prostate CTV, and the bladder was contoured as the whole organ. These clinically approved contours were used as the reference for this study.

CT-based automatic segmentation

Automatic segmentation was performed on the planning CT using OncoStudio (version 2.1.3; Oncosoft Inc., Seoul, South Korea), without any MRI input. The software provides multiple pretrained deep learning models; in this study, the pelvic model (generating contours for the anus, rectum, bladder, and femoral heads) and the prostate model (generating contours for the prostate, seminal vesicles, penile bulb, and the rectal spacer) were used, as automatically selected by the platform for prostate radiotherapy cases. The rectal spacer, prostate, rectum, and bladder were exported from these models for analysis. Auto-contours were used without manual editing, with the following two exceptions specific to the rectum.

First, OncoStudio generates separate contours for the rectum and anal canal. These contours are then merged into a single structure to align with clinical contouring practice, which delineates the rectum and anal canal as one continuous structure. Second, since the superior and inferior extents of the automatic rectum contour did not always align with the reference contour, the slice planes of both contours were aligned at the top and bottom, and any slices outside the reference range were removed to ensure full alignment. This trimming was performed to focus the geometric comparison on the clinically relevant rectal segment adjacent to the target volume, rather than on the entire rectum, because in routine prostate radiotherapy, the rectum is not evaluated as a whole organ but primarily within a limited cranio-caudal extent around the planning target volume. These adjustments were made solely to ensure a fair geometric comparison and did not involve modifying the contour boundaries within the matched slice range.

Evaluation

All contours were quantitatively evaluated against the reference contours. No qualitative visual scoring was performed because the non-radiopaque spacer is not visible on CT, and independently assessing its contour on CT alone is not feasible. Geometric agreement was quantified using the Dice similarity coefficient (DSC), 95th-percentile Hausdorff distance (HD95), and mean surface distance (MSD) [10].

In this study, we also pre-specified geometric criteria for clinical acceptability of the spacer auto-contours, defining “clinically acceptable” performance as the DSC of at least 0.75 and the MSD of at most 2.0 mm. These thresholds were chosen to be consistent with previously reported ranges for human interobserver variability in pelvic structures and with published benchmarks for commercial auto-contouring systems, which typically regard DSC values around 0.8 and distance-based errors of approximately 2-3 mm as clinically acceptable [7,9-12].

Statistical analysis

Agreement between the delineated volumes of the automatic and reference contours was assessed using the mean volume difference (automatic minus reference). A negative value indicates that the automatic contour was smaller than the reference contour. The data were visualized using box plots, which show the median, interquartile range, and individual data points. Given the small sample size and the single-institution feasibility design, the analysis was restricted to descriptive statistics without formal hypothesis testing or confidence interval estimation, and the study was not powered for inferential comparisons or correlation analyses. All analyses and data visualization were performed using Python (version 3.11; Python Software Foundation, Wilmington, DE, USA) with the NumPy, SciPy, and Matplotlib libraries.

## Results

Patient characteristics

The cohort included 21 men with localized prostate cancer who were treated with definitive external beam radiotherapy and a non-radiopaque hydrogel rectal spacer (SpaceOAR). The median age of the patients was 73 years (range: 57-83 years). Patient and treatment characteristics are summarized in Table 1.

**Table 1:** Patient and treatment characteristics (n = 21) Data are presented as median (range) for continuous variables and as number (percentage) for categorical variables.

Characteristic	Value
No. of patients	21
Age, years, median (range)	73 (57–83)
Clinical T stage, n (%)	
T1c	8 (38.1%)
T2a	8 (38.1%)
T2c	3 (14.3%)
T3a	1 (4.8%)
T3b	1 (4.8%)
Gleason score (Grade Group), n (%)	
3+3	2 (9.5%)
3+4	9 (42.9%)
4+3	4 (19.0%)
4+4	6 (28.6%)
Prescription dose/fractionation, n (%)	
60 Gy/20 fx	13 (61.9%)
74 Gy/37 fx	5 (23.8%)
78 Gy/39 fx	3 (14.3%)

Rectal spacer

**Figure 1:**
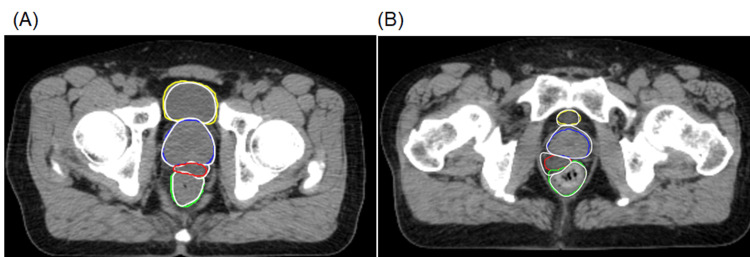
CT-based automatic and reference contours Representative axial planning CT images showing CT-based automatic contours (OncoStudio; colored lines) overlaid with reference contours (white dashed lines) for the rectal spacer (red), prostate (blue), rectum (green), and bladder (yellow). Panel A shows a case with high agreement, in which the automatic and reference spacer contours closely overlap in the retroprostatic space. Panel B shows a case with a larger discrepancy in the spacer region.

Figure 1 illustrates representative examples of CT-based automatic contours overlaid with the reference contours for the rectal spacer, including a case with high agreement and a case with a larger discrepancy in the spacer region. The mean delineated volumes were 11.6 ± 1.6 cm³ for the automatic spacer contour and 11.7 ± 1.8 cm³ for the reference contour, with a mean volume difference (automatic minus reference) of −0.18 ± 2.26 cm³, indicating no systematic over- or under-segmentation (Table 2). The mean DSC between the CT-based auto-contour and the reference was 0.787 ± 0.073, the mean HD₉₅ was 4.03 ± 1.83 mm, and the mean MSD was 1.18 ± 0.63 mm. Lower DSC values were largely due to a few outlier cases (e.g., DSC = 0.631, 0.642, and 0.702), which also had the largest HD₉₅ and MSD values, suggesting localized rather than global discrepancies in delineation.

**Table 2:** Comparison of delineated volumes between CT-based automatic (OncoStudio) and reference contours for all structures (n = 21) Data are presented as mean ± SD. The difference was calculated as the automatic volume minus the reference volume; a negative value indicates a smaller automatic contour volume.

Structure	Automatic volume (cm³)	Reference volume (cm³)	Difference (cm³)
Rectal spacer	11.6 ± 1.6	11.7 ± 1.8	−0.18 ± 2.26
Prostate	35.1 ± 15.7	38.4 ± 18.3	−3.32 ± 4.53
Rectum	56.4 ± 12.5	51.1 ± 13.1	5.29 ± 3.09
Bladder	228.3 ± 92.4	222.6 ± 88.4	5.71 ± 8.37

Adjacent organs

**Figure 2:**
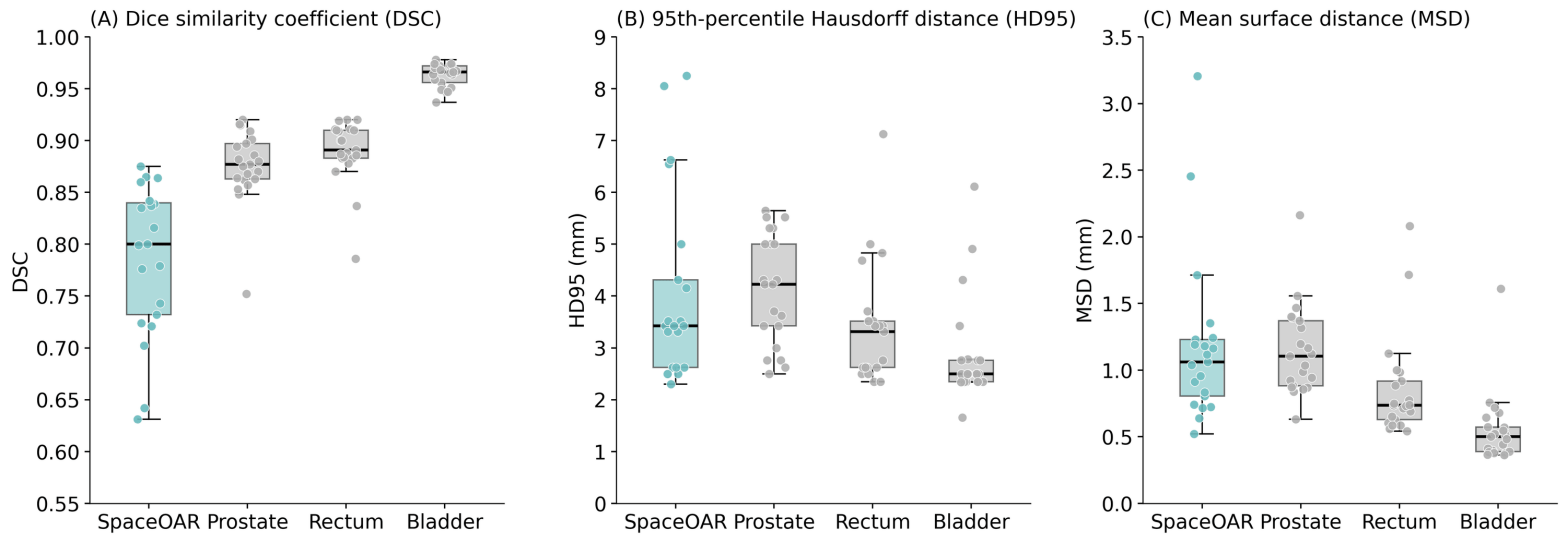
Geometric accuracy metrics for automatic segmentation Distribution of geometric accuracy metrics between CT-based automatic contours (OncoStudio) and the reference contours for the rectal spacer, prostate, rectum, and bladder (n = 21). Panel A shows the Dice similarity coefficient (DSC), panel B shows the 95th-percentile Hausdorff distance (HD₉₅), and panel C shows the mean surface distance (MSD). Box plots display the median and interquartile range; whiskers extend to 1.5 times the interquartile range, and individual data points represent single patients.

The delineated volumes for the prostate, rectum, and bladder showed close agreement between the automatic and reference contours, as summarized in Table 2. The mean DSC was 0.876 ± 0.036 for the prostate, 0.890 ± 0.031 for the rectum, and 0.963 ± 0.011 for the bladder. The corresponding mean HD₉₅ values were 4.15 ± 1.07 mm, 3.41 ± 1.17 mm, and 2.88 ± 1.03 mm, and the mean MSD values were 1.15 ± 0.34 mm, 0.86 ± 0.38 mm, and 0.55 ± 0.27 mm, respectively. The MSD was below 2.5 mm for all patients and all three organs. The geometric accuracy metrics for all four structures are summarized in Table 3, and their distributions are shown in Figure 2.

**Table 3:** Geometric accuracy of CT-based automatic segmentation (OncoStudio) compared with reference contours for all four structures (n = 21) Data are presented as mean ± standard deviation. DSC, Dice similarity coefficient; HD₉₅, 95th-percentile Hausdorff distance; MSD, mean surface distance.

Structure	DSC	HD₉₅ (mm)	MSD (mm)
Rectal spacer	0.787 ± 0.073	4.03 ± 1.83	1.18 ± 0.63
Prostate	0.876 ± 0.036	4.15 ± 1.07	1.15 ± 0.34
Rectum	0.890 ± 0.031	3.41 ± 1.17	0.86 ± 0.38
Bladder	0.963 ± 0.011	2.88 ± 1.03	0.55 ± 0.27

## Discussion

This study examined the use of a commercial deep learning platform (OncoStudio) for CT-based automatic segmentation of a non-radiopaque hydrogel rectal spacer. This approach achieved geometric accuracy that met our pre-specified criteria for clinical acceptability in the majority of patients, without MRI input. Although the DSC for the spacer (0.787 ± 0.073) was relatively lower than those of the prostate, rectum, and bladder, the distance-based metrics showed favorable agreement. Notably, the boundary agreement of the spacer (MSD, 1.18 mm) was comparable in absolute terms to that of the prostate (MSD, 1.15 mm), although we recognize that for a much smaller structure such as the spacer, a similar absolute surface distance corresponds to a proportionally larger relative boundary error. No systematic degradation of the prostate or rectum contours was observed in the region adjacent to the spacer. The prostate, rectum, and bladder also showed high agreement with the reference, with mean DSCs of 0.876, 0.890, and 0.963, respectively, comparable to previously reported values for OncoStudio and other deep learning platforms [9,11-13]. For the rectum, these metrics reflect agreement within the clinically relevant segment surrounding the target rather than along the entire rectal length, and thus likely overestimate agreement if the full rectum were considered. These findings indicate that accurate spacer segmentation can be achieved without compromising the contouring quality of surrounding organs.

In our cohort, three cases showed notably lower spacer DSC values (0.631, 0.642, and 0.702) with correspondingly larger HD₉₅ and MSD, indicating localized disagreement between the automatic and reference contours. From a clinical perspective, these outliers may represent potential failure modes in which CT-only auto-contouring would be insufficient and MRI-based spacer delineation or review would still be required. Furthermore, because the non-radiopaque spacer is not directly visible on CT, a failed auto-contour cannot be visually verified at the point of care once MRI has been omitted. Any implementation of a CT-only workflow should therefore incorporate safeguards or criteria to identify such cases and prompt MRI-based spacer reassessment or additional quality assurance measures.

This result contrasts with earlier studies on CT-based spacer segmentation. Wang et al. developed separate deep learning models for the non-radiopaque and radiopaque spacer formulations; however, the CT-based model for the non-radiopaque spacer had limited clinical value, with a mean DSC of only 0.52, and its inaccuracy degraded the contour quality of the adjacent prostate and rectum [7]. Therefore, MRI has been considered essential for spacer delineation, with T2-weighted imaging regarded as the reference standard. Although the DSC achieved in this study was moderate, it represents a clear improvement over the earlier CT-based attempt, and our results suggest that a contemporary commercial platform can partially overcome the challenges of delineating a non-radiopaque spacer on CT alone. However, clinical benefit for a rectal spacer is ultimately defined by dosimetric endpoints (rectal dose reduction and toxicity), and DSC, HD₉₅, and MSD are geometric surrogates rather than direct clinical outcomes. In this feasibility study, “clinical acceptability” therefore refers only to the pre-specified geometric thresholds and should be interpreted as hypothesis-generating rather than confirmatory.

In current clinical workflows, the non-radiopaque spacer is typically contoured on MRI and transferred to the planning CT through image registration, a step that inevitably introduces spatial uncertainty into the planning geometry. Direct CT-based delineation avoids the additional registration step required to transfer MRI-derived spacer contours to the planning CT and may reduce registration-related spatial uncertainty, while extending applicability to patients who cannot undergo MRI because of contraindications such as implanted devices or severe claustrophobia. However, because our reference contours were themselves derived from MRI-CT registration, this study primarily demonstrates that the model can reproduce MRI-informed spacer boundaries on CT rather than establishing a registration-independent anatomical ground truth. This does not imply, however, that MRI becomes unnecessary in prostate radiotherapy as a whole, since MRI retains a central role in tumor staging, target definition, and prostate delineation.

Several methodological considerations should be noted regarding spacer evaluation. Because the non-radiopaque spacer is nearly isodense on CT, qualitative visual assessment of its contour is not feasible, and evaluation relied solely on quantitative metrics. The DSC is intrinsically penalized for small, variably shaped structures, as a minor boundary discrepancy translates into a disproportionately large reduction in overlap; this explains why the spacer showed a relatively low DSC despite an MSD comparable to that of the prostate.

This study has several limitations. First, it was a single-institution retrospective analysis with a limited sample size, and validation in larger, multi-institutional cohorts is warranted. Second, the reference contour for the spacer was derived from MRI and transferred to the planning CT through image registration; consequently, the reference itself incorporates registration-related uncertainty, and any discrepancy between the automatic and reference contours cannot be attributed solely to CT-based segmentation. This represents an inherent asymmetry when evaluating a structure that is not directly visible on CT. Third, although all reference contours were reviewed and approved by a senior radiation oncologist, initial delineations were performed by different radiation oncologists, which may have introduced interobserver variability. Interobserver variability was not formally quantified for the spacer in this study; therefore, the model’s performance cannot be directly compared with human interobserver agreement for this structure. Another limitation is that OncoStudio is a commercial deep learning platform. The specific model architecture, training data composition, and versioned weights of the pelvic and prostate models are not publicly disclosed, and users cannot modify or retrain them. As a result, our results are inherently platform-specific, and independent replication of the exact model behavior is not possible without access to the same licensed software and model version. In particular, the spacer formulations included in the training data (radiopaque vs non-radiopaque) are unknown, which introduces additional uncertainty when generalizing our findings to other spacer formulations. Furthermore, because this was a small, single-institution feasibility study with purely descriptive statistics and no dosimetric endpoints, our findings should be interpreted as hypothesis-generating rather than confirmatory, and the lack of inferential testing and dose-based validation limits the strength of any comparative claims. Future studies with larger, multi-institutional cohorts and prospective designs are warranted to validate these findings and to assess the clinical impact of CT-only spacer delineation on dosimetric outcomes.

## Conclusions

CT-based automatic segmentation using a commercial deep learning platform (OncoStudio) provided clinically acceptable boundary delineation of a non-radiopaque hydrogel rectal spacer without requiring MRI, while maintaining the contouring quality of the surrounding organs. Although the DSC overlap for the spacer was moderate, the distance-based agreement approached that of the adjacent prostate, indicating good boundary conformity. These results suggest that CT-only automatic contouring may reduce the need for spacer-specific MRI and the associated registration step in prostate radiotherapy, but prospective validation including dosimetric endpoints and robust QA procedures is required before CT-only workflows can be adopted routinely.
